# Usability and Vibration Analysis of a Low-Profile Automatic Powered Wheelchair to Motor Vehicle Docking System

**DOI:** 10.3390/vibration6010016

**Published:** 2023-02-24

**Authors:** Chang Dae Lee, Brandon J. Daveler, Jorge L. Candiotti, Rosemarie Cooper, Sivashankar Sivakanthan, Nikitha Deepak, Garrett G. Grindle, Rory A. Cooper

**Affiliations:** 1 Human Engineering Research Laboratories, Department of Veterans Affairs Pittsburgh Healthcare System, University of Pittsburgh, Pittsburgh, PA 15206, USA; 2 Department of Rehabilitation Science and Technology, University of Pittsburgh, Pittsburgh, PA 15213, USA; 3 Department of Bioengineering, University of Pittsburgh, Pittsburgh, PA 15213, USA

**Keywords:** accessible transportation, wheelchair docking system, assistive technology, securement, vehicle safety

## Abstract

The QLX is a low-profile automatic powered wheelchair docking system (WDS) prototype developed to improve the securement and discomfort of wheelchair users when riding in vehicles. The study evaluates the whole-body vibration effects between the proposed QLX and another WDS (4-point tiedown system) following ISO 2631-1 standards and a systematic usability evaluation. Whole-body vibration analysis was evaluated in wheelchairs using both WDS to dock in a vehicle while riding on real-world surfaces. Also, participants rated the usability of each WDS while driving a wheelchair and while riding in a vehicle in driving tasks. Both WDSs showed similar vibration results within the vibration health-risk margins; but shock values below health-risk margins. Fifteen powered wheelchair users reported low task load demand to operate both WDS; but better performance to dock in vehicles with the QLX (*p* = 0.03). Also, the QLX showed better usability (*p* < 0.01), less discomfort (*p’*s < 0.05), and greater security compared to the 4-point tiedown while riding in a vehicle (*p’*s < 0.05). Study findings indicate that both WDS maintain low shock exposure for wheelchair users while riding vehicles, but a better performance overall to operate the QLX compared to the 4-point tiedown system; hence enhancing user’s autonomy to dock in vehicles independently.

## 1. Introduction

In 2021, the American Community Survey (ACS) determined that 13% of the population in the United States has a disability [1]. The highest prevalence of disability of the six categories identified by the ACS was having an ambulatory disability at 6.6%, which increased significantly with age. Also, the 2017 National Household Travel Survey found that 25.5 million people had disabilities that limited their ability to travel, of which 20% use wheeled mobility devices, including manual wheelchairs, powered wheelchairs, and scooters [2]. For such individuals, a reliable method of transportation is necessary to work, attend school, or actively participate in the community. Public transportation may be insufficient in some communities due to limited flexibility. Paratransit or accessible taxis may require scheduling ahead of time; thus, impromptu use of these services may not be possible. Furthermore, these limitations are exacerbated for those who live in suburban and rural areas where wheelchair users are highly dependent on personal vehicles [3].

Manual wheelchair users can transfer to a vehicle seat to drive or ride as a passenger in a personal vehicle; meanwhile, a wheelchair docking system (WDS) is essential to secure a power wheelchair user within the vehicle [4]. A WDS is an interface between a motor vehicle and a wheelchair that secures the wheelchair to the vehicle to facilitate the safe use of the wheelchair as a seat in a motor vehicle [4]. All WDS serve the same purpose to provide security and safety by firmly securing the wheelchair to the vehicle. The 4-point tiedown (4-TD) system is widely used because it is more affordable and flexible than most other WDS; however, wheelchair users require assistance to use them [5–7]. Automatic WDS allows wheelchair users to secure their wheelchairs quickly and independently [8]. It uses a docking pin installed on the bottom of the wheelchair to lock it on a system installed on the vehicle floor [4,9]. On the other hand, it reduces the wheelchair’s ground clearance making it prone to hit uneven surfaces [4,10], resulting in injuries to the user and wheelchair damage [11]. Alternatively, BraunAbility and Q’Straint developed the prototype of a low-profile automatic WDS called QLX. The study aimed to evaluate the QLX in terms of usability, task load, comfort, and securement compared to other WDS.

The second goal of the study was to examine the vibration transmissibility of WDS while riding in a vehicle. Typically, the human body is regularly exposed to whole-body vibration (WBV), particularly while traversing rough and uneven surfaces. WBV exposure is exacerbated in vehicle drivers and passengers exposed for a long period of time that reach moderate to high health risk per ISO 2631-1 [12–15]. This exposure increases discomfort and the risk of muscle fatigue, pain, connective nerve damage, and spinal disorders [16–18]. Compared to vehicle seats, wheelchair users require a WDS to be secured in a vehicle which may affect the WBV effects and discomfort while riding a vehicle. Van Roosmalen et al. [6,8] evaluated wheelchair users’ level of discomfort using different WDS in a transit vehicle. However, to the author’s knowledge, the WBV exposure and discomfort of wheelchair users while driving or riding a vehicle is unclear. WBV exposure and discomfort while using a WDS should be investigated when considering its impact on the health conditions and mobility of wheelchair users. To fill the gaps, we will examine the WBV effects in power wheelchair users using different WDS in a vehicle. The study aimed to: 1) compare the WBV exposure between two WDS (i.e., QLX and 4-TD system) while riding in a vehicle over real-world surfaces (WBV test); 2) perform a systematic usability evaluation of the QLX compared to participants’ WDS while performing controlled wheelchair driving tasks (wheelchair driving test); and 3) perform a systematic usability evaluation of the QLX compared to participant’s WDS while riding in a vehicle through a controlled driving course (vehicle riding test).

## 2. Materials and Methods

### 2.1. Instrumentation

#### 2.1.1. Wheelchair Docking Systems

**Figure 1.**
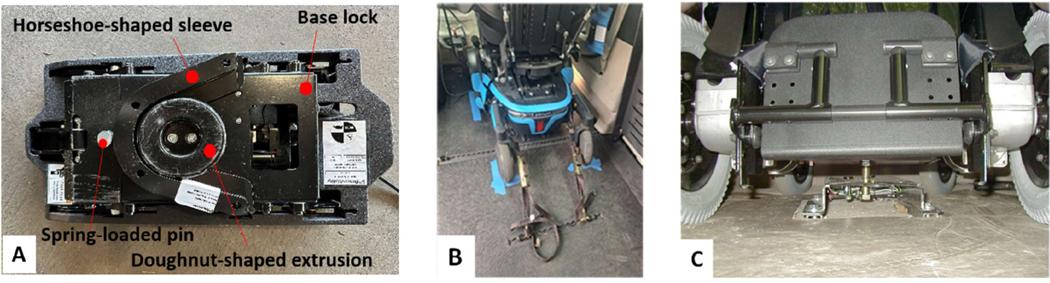
**(A)** QLX WDS, **(B)** 4-point tiedown system, **(C)** EZ-Lock.

The QLX WDS prototype consists of 2 core interlocking components accompanied by a touchscreen graphical user interface and manual release handle. The interlocking components include the ″base lock″ mounted to the vehicle chassis and ″the horseshoe-shaped sleeve″ mounted to the wheelchair undercarriage. The ″base lock″ uses a linear actuator and scissor mechanism with a doughnut-shaped extrusion designed to connect with the ″horseshoe-shaped sleeve.″ When the wheelchair drives over the ″base″, there is a spring-loaded pin that is pushed down by the weight of the wheelchair to then dock with the ″base″. Between the ″base″ and ″horseshoe-shaped sleeve″, there is an electromagnetic sensor that identifies when the wheelchair is docked and aligned correctly before signaling a prompt to anchor down the wheelchair. At this point, the ″base plate″ will lower, thus anchoring the wheelchair, and then the spring-loaded pin has a small actuator that will lock the pin in place, stabilizing the wheelchair. In the event of an emergency, there is a manual release handle that will pull down the spring-loaded pin to allow the wheelchair user to exit the WDS (Figure 1A). The QLX was attached to a commercially available power wheelchair (Model: Permobil F3) for the study. Participants’ own WDS included a 4-TD system or an EZ-Lock. The 4-TD system, as required by WC-19 wheelchair standards, uses 4 attachment points located in each corner of a power wheelchair to secure it with straps to the floor of the vehicle (Figure 1B). The EZ-Lock is an automated docking system using a pin installed under the wheelchair that locks to a docking system installed in the vehicle (Figure 1C).

#### 2.1.2. Whole-Body Vibration Monitoring

**Figure 2.**
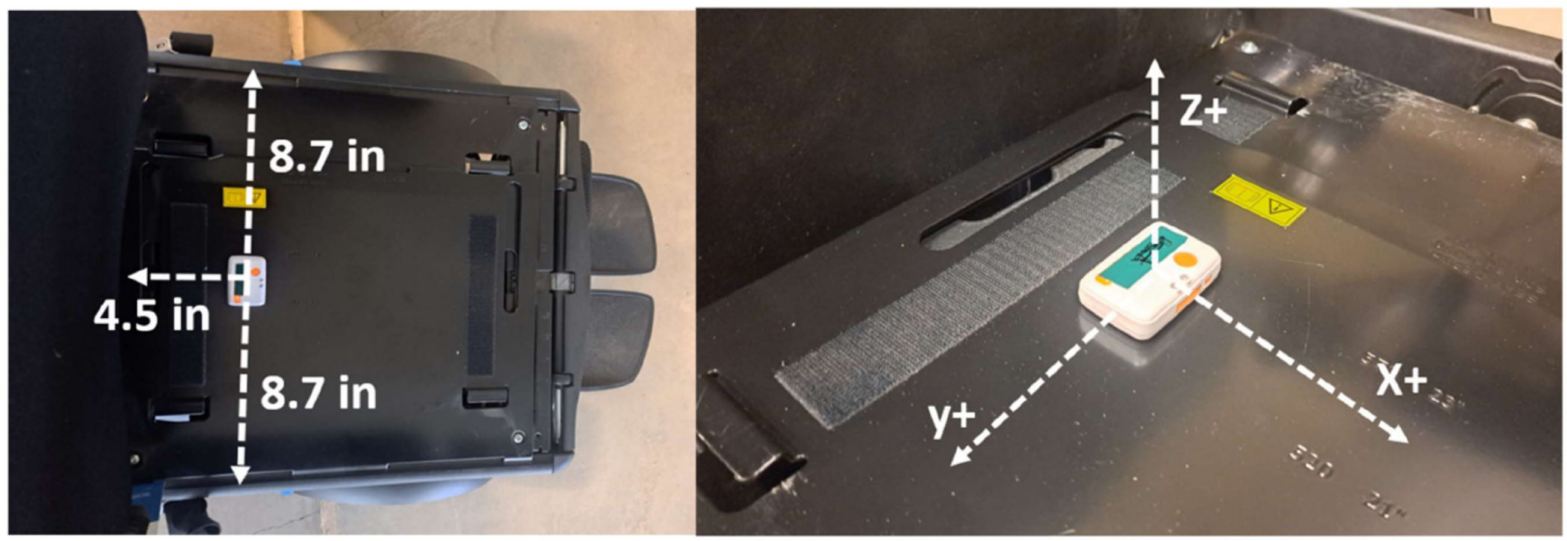
Location (**Left**) and orientation (**Right**) of the Shimmer 3 on the wheelchair.

We used the Shimmer 3 (Shimmer, Dublin, Ireland), a triaxial accelerometer connected to a stand-alone microcontroller (STMicro LSM303AHTR) with a 14-bit resolution to detect +/−8 g and at a sampling rate of 100 Hz. The Shimmer sensor was located under a rehabilitation seating cushion to measure vibrations transmitted to wheelchair users with its z+ axis facing orthogonal to the floor of the vehicle (Figure 2) [17]. A sampling frequency of 100 Hz was selected to identify a suitable range of frequencies between 0.01 and 80 Hz according to ISO 2631-1 (1997) standard [15]. The sensor was fixed on the seat pan to prevent unwanted movement during the testing. Root mean square (RMS) and vibration dose values (VDV) were calculated from acceleration data following ISO 2631-1.

### 2.2. Participants

Aim 1 (WBV Test) was performed with an anthropomorphic test dummy, Rescue Randy^®^ (a 6-foot-l-inch-tall dummy weighing 165 pounds), to avoid the risk of potentially injuring the participants or exposing them to discomfort. Aim 2 (wheelchair driving test) and Aim 3 (vehicle riding test) involved subject testing. Inclusion criteria included: (1) be 18 years old or older; (2) use a power wheelchair as their primary means of mobility; (3) agree to take a test ride with a wheelchair in a vehicle; and (4) be able to provide written informed consent in the English language. Participants were excluded if they: (1) had a current or recent history of pressure injuries that could be exacerbated by prolonged sitting; (2) were unable to transfer to or drive from a study wheelchair provided for them; and (3) were unable to provide up to 4 h to participate in study testing. The study was approved by the University of Pittsburgh Institutional Review Board (IRB; STUDY22080113).

### 2.3. Experimental Setup

#### 2.3.1. Wheelchair Driving Course

**Figure 3.**
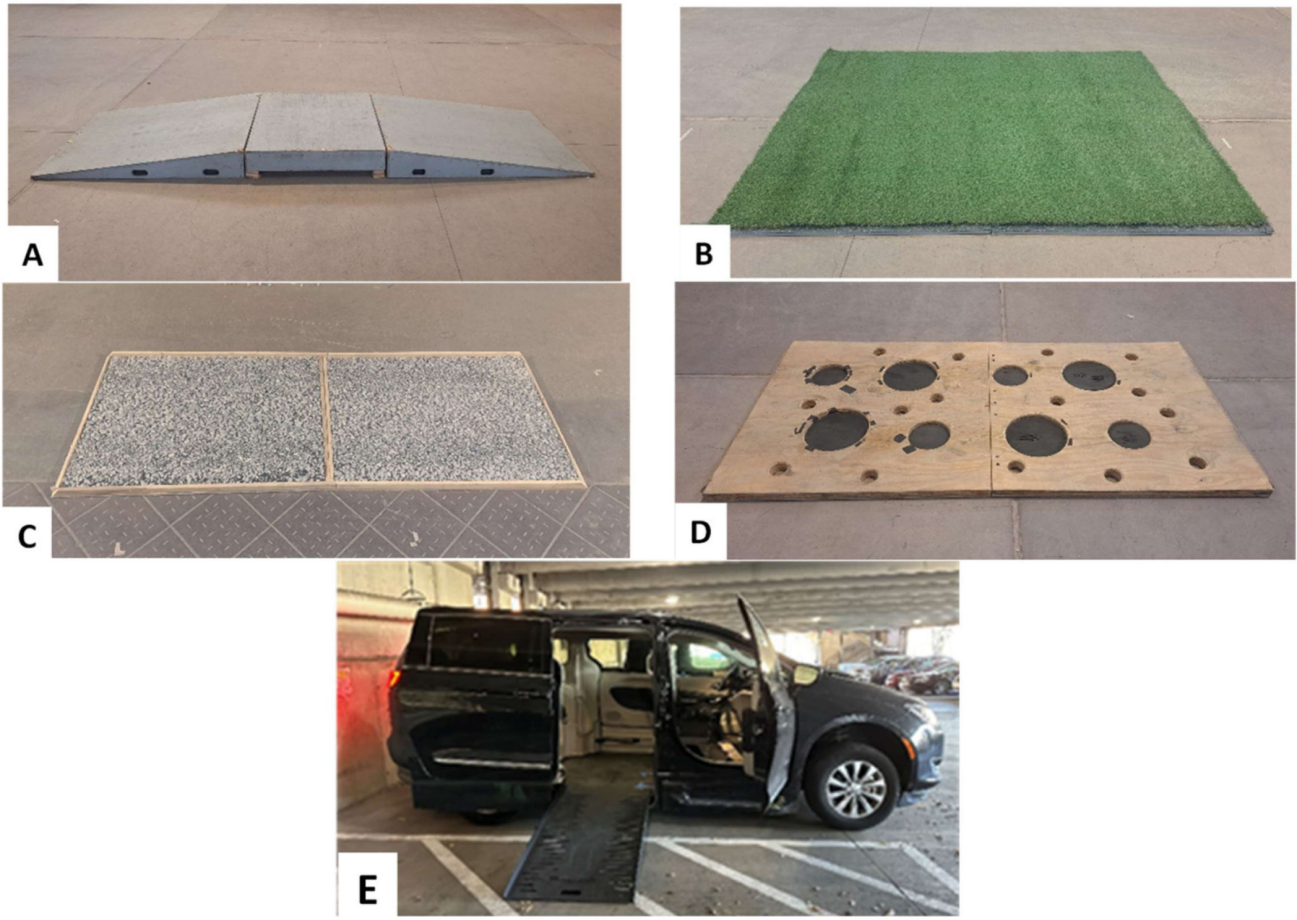
Wheelchair test course. (**A**) Curb cut, (**B**) Grass, (**C**) Gravel, (**D**) Potholes 1″ depth, (**E**) Accessible vehicle.

The wheelchair driving course included a curb cut, grass, uneven surface, pothole, accessible vehicle ingress/egress (Figure 3A-E) and docking in the vehicle using a WDS. The curb cut consisted of 5° up and down slopes with a transition surface. The grass task was 8.0 ft long × 6.0 ft wide of artificial turf (Make: TrafficMaster) installed on top of 3/4 ″ soft rubber tiles to simulate real grass. The uneven surface simulated driving on a gravel road (8′ long × 6′ wide). The pothole task used a plywood platform (8′ long × 4′wide) with several 1-inch-deep potholes of up to 12 inches in diameter. The ingress/egress task used a power ramp deployed by a study vehicle (Model: 2019 Chrysler Pacifica, modified by Braun Ability).

#### 2.3.2. Vehicle Riding Course

**Figure 4.**
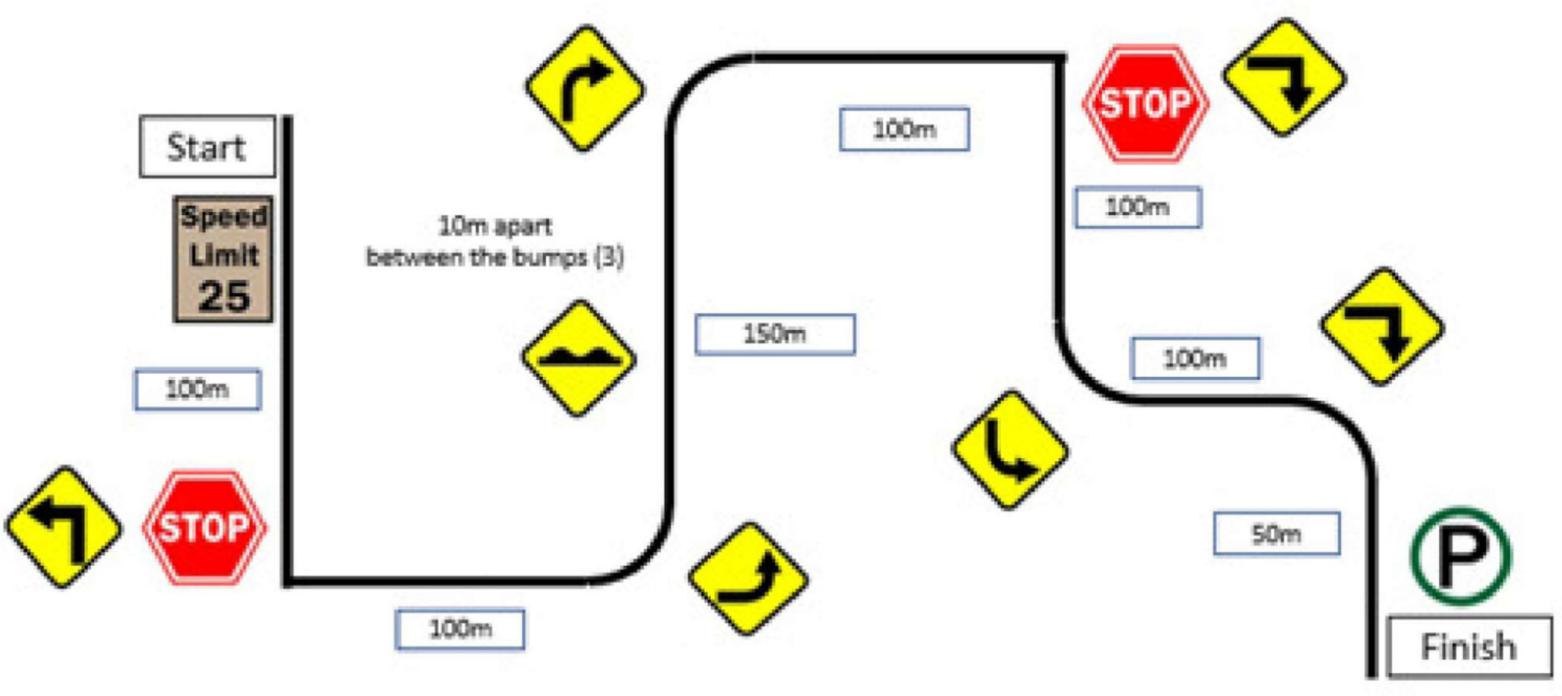
Vehicle riding test course.

The vehicle riding test course included 3 left/right turns, 2 stop signs, and 3 consecutive speed bumps (Figure 4). Per IRB requirements, driving speed did not exceed 25 mph.

#### 2.3.3. Vibration Test Obstacle Course

**Figure 5.**
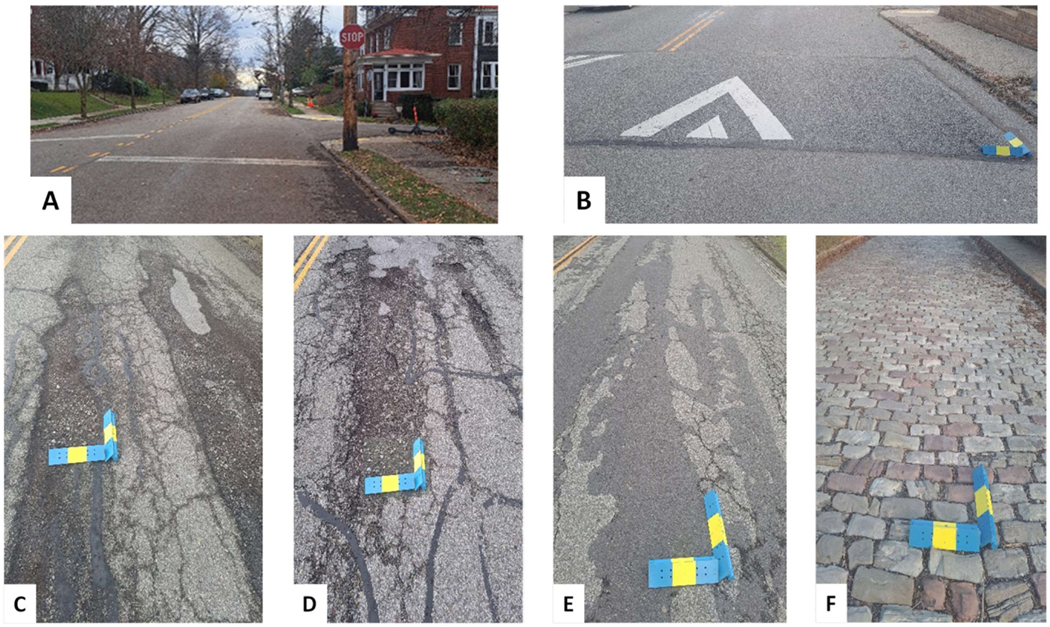
Vibration tests driving tasks: (**A**) Sudden Brake, (**B**) Speedbump, (**C**) Pothole 1, (**D**) Pothole 2, (**E**) Uneven surface, (**F**) Cobblestone up/down hill. Blue/yellow object 16-inch-long for scale.

The vibration tests were performed with 7 different driving tasks: sudden brake, potholes 1 and 2, speed bump (3.8″ height × 11.5′ wide), uneven surface, and cobblestone up/downhill (Figure 5). Potholes 1 and 2 consist of sets of potholes that were formed naturally in asphalt pavement roads. Pothole 1 was a total 50′ long surface with multiple pothole dimensions of up to 18′ long x 3.8′ wide. Pothole 2 was a total 42′ long surface with multiple potholes, including 6.8′ long x 2′ wide, 5′ long x 1.2′ wide, 1.9′ long x 2.6′ wide, and smaller potholes. An uneven surface consists of an asphalt pavement road with a height difference between the original and the repaired road surface (265′ long). The cobblestone up/downhill was a total 400′ long with an approximate slope of 9.2°. The average size of cobblestone was 10.4″ long × 4.4″ wide × 0.4″ height above the surface plane. All vibration tests were conducted on public roads in low-traffic zones, following traffic laws and driving rules in the area.

### 2.4. Procedure

Aim 1—WBV test: A study wheelchair using the QLX prototype was docked to a study vehicle, and then the same wheelchair was docked using a 4-TD (See Section 2.1). An anthropomorphic test dummy (Rescue Randy^®^, 6-foot-l-inch-tall dummy weighing 165 pounds) was used to prevent any risk of injuring or causing discomfort to wheelchair users. The test dummy was seated in a 90–90-90 position and seat belted into the wheelchair to control its movement. Later, a designated research team member drove the vehicle through the vibration test obstacle course for 5 trials with the QLX prototype and the 4-TD for a total of 70 trials. The vehicle’s cruise control function was set to approximately 25 mph to maintain a consistent speed for each trial, except for tasks such as speed bumps (15 ± 2 mph, obeying traffic laws) and cobblestone up/downhill (10 ± 2 mph, determined by comfort of the vehicle driver). The target speed was reached at least 3 seconds before passing each driving task. The WBV measurements of the wheelchair seat were collected starting 3 seconds before each driving task and for 3 s after. The wheelchair was positioned on the passenger side, and its location was marked to confirm that the 2 securement systems were engaged in the same position in the vehicle (Figure 1B). The QLX prototype was removed from the vehicle to provide ground clearance for the test wheelchair using the 4-TD.

**Figure 6.**
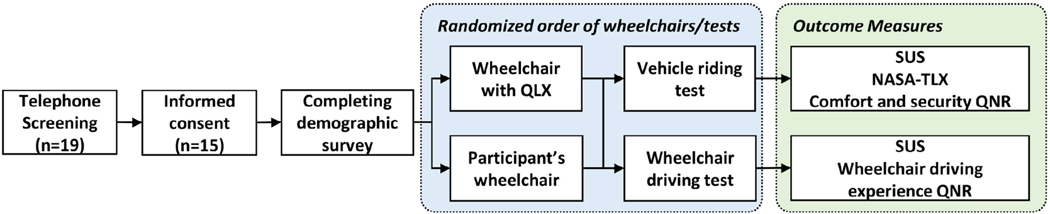
A flowchart for the wheelchair driving and vehicle riding tests procedure.

Aim 2 and 3—Wheelchair Driving and Vehicle Riding Tests: The usability evaluation of WDS in wheelchair driving (Aim 2) and vehicle riding (Aim 3) tests involved subject testing and was conducted at the Pittsburgh International Race Complex in Wampum, PA. The duration of the study was one visit up to 4 h long. Informed consent was obtained from participants upon their arrival at the study site. Participants were asked to complete a brief demographic survey (e.g., gender, ethnicity, age, disability etiology, driving experience, etc.). Later, participants were randomized in the order of wheelchair use (study wheelchair with QLX or wheelchair with own WDS) and tests (wheelchair driving or vehicle riding) to limit the wheelchair transfers to only 2 (Figure 6). Commercial power wheelchairs typically include a 4-TD to meet WC-19 standards [5]. Alternatively, wheelchair users may own an automatic WDS (e.g., EZ-Lock). Preceding testing, the study wheelchair with QLX was set up to the participant’s wheelchair driving and seating configurations. Participants were allowed to use their personal seat cushions if desired, and body supports were provided if needed. After transferring to the study wheelchair, participants received training and practiced using the device for about 15 min without difficulty as they drove a power wheelchair on a daily basis.

Aim 2—Wheelchair driving test: Participants were asked to complete the wheelchair driving course 3 times using the study wheelchair with QLX and their own wheelchair with WDS. During the docking task, if participants had a wheelchair-accessible vehicle, they were allowed to use their own wheelchair docking system (e.g., 4-TD or EZ-Lock) in their own vehicles. Otherwise, participants were asked to use their 4-TD in the study vehicle, where research team members engaged the tiedown system to participants’ wheelchairs. Upon completion of the third trial, participants were asked to complete the System Usability Scale (SUS) and a customized wheelchair-driving experience questionnaire.

Aim 3—Vehicle Riding Test: Participants were asked to enter the vehicle and dock the assigned wheelchair (QLX or own wheelchair with 4-TD/EZ-Lock) in the passenger side of the vehicle. After ensuring that the wheelchair was properly docked, a designated research team member drove the vehicle with the participant through the vehicle riding test course 3 times. If participants wanted additional tests, they could ride up to 6 times. The same person drove the vehicle at a constant speed of 25mph to control and minimize unexpected factors. The process was repeated with the other wheelchairs. If participants had their personal vehicle, participants were asked to dock with their wheelchair in their vehicle and would drive the course or ride the course with their driver. Upon completion of the third trial with each wheelchair, participants were asked to complete the NASA Task Load Index (NASA TLX), SUS, and comfort and security questionnaires.

### 2.5. Measures of Usability

#### 2.5.1. System Usability Scale (SUS)

The SUS has been widely used for testing the usability of products and services, including devices, websites, software, and hardware [19]. In this study, the SUS was used to measure the usability of the QLX and the participant’s WDS. The SUS consists of 10 questions (including ‘I think that I would like to use this system frequently’, ‘I found the system unnecessarily complex’, ‘I thought the system was easy to use’, and ‘I think that I would need the support of a technical person to be able to use this system’) and uses a 5-point Likert scale (1 = strongly disagree, and 5 = strongly agree) [19]. To score the SUS, subtract one from the user responses for odd items and subtract the user response from 5 for even items. Then, add up the scores obtained for each item (original score range 0 to 40) and multiply the result by 2.5 to create a converted score with a range from 0 to 100. A higher score indicates better usability, with a minimum of 69 to be considered acceptable. SUS is a reliable tool, and its coefficient alpha was α = 0.92 [20].

#### 2.5.2. NASA Task Load Index (NASA TLX)

The NASA TLX has been widely used to measure subjective workload [21]. In this study, NASA TLX was used to assess the workload of study participants when operating and using the QLX and participants’ WDS, respectively. The NASA TLX consists of 6 domains, including mental demand, physical demand, temporal demand, performance, effort, and frustration. The NASA TLX has good reliability, and the test-retest reliability was 0.77 [22]. The validity of NASA TLX has been established by comparing it with the other 2 workload measurements, construct validity was 0.97 and 0.98, and concurrent validity was 0.73 and 0.79 [23].

#### 2.5.3. Wheelchair Driving Experience Questionnaire

A study-specific questionnaire was developed to additionally measure the usability of the QLX and the participant’s WDS (Table S1). The questionnaire consisted of 7 items. Four items asked about the experience of driving a wheelchair over various surfaces (including curb cuts, grass, uneven sidewalk, and potholes), 2 items asked about the experience of using a WDS (including entering/exiting the vehicle and docking in the vehicle), and one item asked overall experience of using a wheelchair. The questionnaire used an 11-point Likert scale (0 = bad experience, 5 = neutral experience, and 10 = good experience), and thus each item score ranges from 0 to 10.

#### 2.5.4. Comfort and Security Questionnaires

Comfort (Table S2) and security questionnaires (Table S3) were developed for this study to assess riding a vehicle using the QLX and their current approach. The comfort questionnaire consists of 4 items asking how comfortable participants felt when (1) riding in the vehicle, (2) docking in the vehicle, (3) entering/exiting the vehicle, and (4) overall. The security questionnaire consisted of 5 items asking how secure participants felt when (1) the vehicle was accelerating/starting, (2) the vehicle was decelerating/stopping, (3) the vehicle was turning, (4) riding in the vehicle, and (5) overall. Both questionnaires used an 11-point Likert scale (0 = Not at all comfortable/secure, 5 = moderately comfortable/secure, and 10 = Extremely comfortable/secure), and thus each item score ranged from 0 to 10.

### 2.6. Statistical Analysis

Descriptive statistics and bar graphs were used to analyze participant demographic information and outcome measures (e.g., means, standard deviation, median and interquartile ranges (IQR)). Paired *t*-tests were used to compare the WBV exposure between QLX and 4-TD while riding a vehicle on real-world surfaces (Aim 1). The WBV was evaluated in terms of total RMS and VDV. The RMS and VDV values for each axis were calculated using the raw acceleration data from the triaxial accelerometer. The raw acceleration data $\left(a_{x},a_{y},a_{z}\right)$ were first calibrated by subtracting the first acceleration value of each axis from its corresponding value. Then the values were multiplied by their frequency weighting in terms of comfort $\left(k_{x}=ky=k_{z}=1\right)$. The study focused on vibration values in the seat pan because the backrest and footplate were fixed to the seat, and we considered the seat as a rigid body. The first and last 3 s timestamps of the data were cut-off to analyze the corresponding driving task. Then, the total magnitude of each acceleration of the 3 axes was determined (Equation (1)) and used to calculate total RMS and total VDV values (Equations (2) and (3)) using MATLAB R2022b [24]. The mean RMS and VDV values of all trials between docking systems were calculated and compared with a within-subjects paired *t*-test analysis using Microsoft Excel.

(1) $$a_{Total}={\left(k_{x}^{2}a_{x}^{2}+k_{y}^{2}a_{y}^{2}+k_{z}^{2}a_{z}^{2}\right)}^{\frac{1}{2}}$$

(2) $$RMS={\left[\frac{1}{T}\int_{0}^{T}a_{Total}{\left(t\right)}^{2}dt\right]}^{\frac{1}{2}}$$

(3) $$VDV={\left[\frac{1}{T}\int_{0}^{T}a_{Total}{\left(t\right)}^{4}dt\right]}^{\frac{1}{4}}$$

where *T* was the duration of each driving task.

Wilcoxon signed ranks test was used to compare the mean scores of the SUS, NASA TLX, wheelchair driving experience questionnaire, and comfort and security questionnaires between the QLX and participant’s own WDS during the wheelchair driving test (Aim 2) and vehicle riding test (Aim 3). All statistical analyses, except for RMS and VDV values and paired *t*-tests, were performed using Stata 16 [25]. The significance level for all analyses was set at 0.05.

## 3. Results

**Figure 7.**
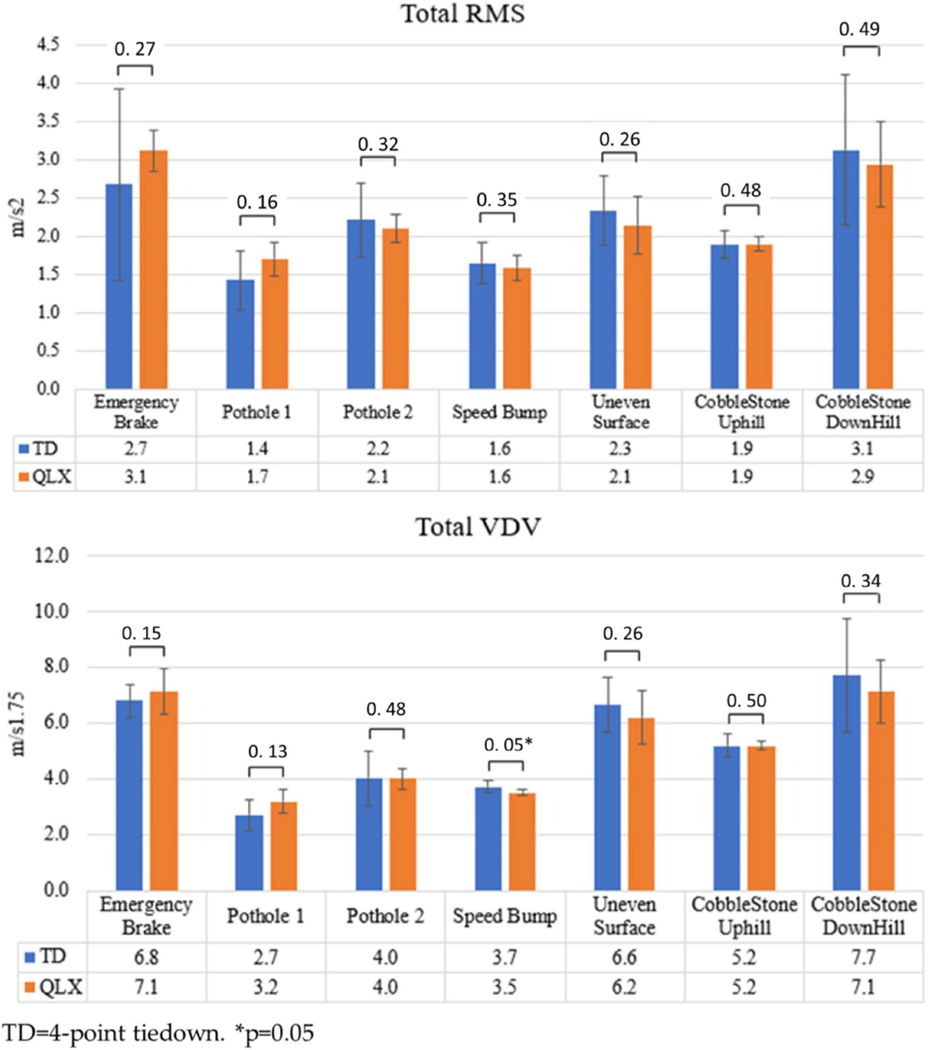
Whole-body exposure analysis of 4-TD and QLX wheelchair docking systems.

Vibration test results: The weighted RMS and VDV values of the QLX and 4-TD when riding a vehicle on different driving tasks are shown in Figure 7. WBV analyses did not reveal statistically significant differences between the 4-TD and QLX except when driving over a speed bump. In this case, the QLX (3.5 ± 0.1 m/s^1.75^) had a significantly lower VDV value than the 4-TD (3.7 ± 0.2 m/s^1,75^; *p* = 0.05).

Study participants: Nineteen powered wheelchair users were scheduled to participate in this study, but four of them did not arrive at the study site. As such, 15 powered wheelchair users participated in the study. The demographics of participants are presented in Table 1. Seven of 15 (46.7%) were female, and most of the participants were White or Caucasian (n = 13; 86.7%) and aged between 35 and 54 (n = 8,53.3%). Three participants reported having an EZ-Lock, and 12 participants had 4-TD.

Wheelchair driving test results: Participants reported no significant differences in usability (SUS) between the QLX prototype and their own WDS (4-TD and EZ-Lock) (Table 2). With regard to wheelchair driving experience, participants reported better experience at docking a wheelchair using the QLX prototype in the vehicle compared to using their WDS (*p* < 0.05). Similar results were shown if EZ-Lock users’ responses (n = 3) were excluded from the analysis.

Vehicle riding test results: Participants reported higher SUS scores using the wheelchair with QLX compared to their own WDS (4-TD and EZ-Lock) (*p* < 0.001) (Table 3). However, participants reported no significant differences in NASA TLX results between the QLX and both WDS. The comfort questionnaire results showed that participants felt significantly more comfortable with the QLX compared to participants’ WDSs for all vehicle riding tasks (all *p*’s < 0.05). The security questionnaire showed that participants reported feeling more secure riding in a vehicle with the QLX compared to participants’ WDS for all vehicle riding tasks (all *p*’s < 0.5), except when starting and accelerating the vehicle.

When responses of participants who did not use 4-TD were excluded (Table S4), there were no significant differences in SUS scores between QLX and 4-TD (*p* = 0.24). However, participants reported significantly less effort to operate the QLX compared to the 4-TD (*p* < 0.05). The comfort questionnaire results showed that participants felt more comfortable docking in the vehicle with QLX compared to the 4-TD (*p* = 0.03). The security questionnaire showed that participants felt more secure riding in a vehicle with the QLX compared to the 4-TD for all vehicle riding tasks (all *p*’s < 0.5), except when starting/accelerating and decelerating/stopping the vehicle.

## 4. Discussion

The study examined WBV exposure with different docking systems (QLX and 4-TD) while riding in a vehicle using a test dummy. The QLX reduced the vibration exposure and shocks slightly better than the 4-TD, most likely due to the rigidity of the QLX and the amount of play in 4-TD when using the straps. The shock reduction with QLX was significantly better than the 4-TD while driving over speed bumps. However, the results showed no statistical differences between both QLX and 4-TD systems across all driving tasks. When comparing the WBV exposure of each WDS with the health guidance caution zone in ISO 2631-1:1997; it was shown that their RMS were within the health-risk zone for a 30-minute exposure (range: 1.5–3.0 m/s^2^). These values are related to the user’s discomfort and potential health effects if exposed for a long period of time. On the other hand, participants reported higher comfort and security scores with QLX compared to their personal WDS while riding the vehicle. A potential explanation of this inconsistency is that the WBV exposure time during the vehicle riding tests was less than the ISO health guidance; therefore, tolerable to wheelchair users. WBV exposure may have been influenced by external factors not related to the WDS, such as vehicle suspension, speed, and driving style, meaning that the securement of the WDS may not increase nor reduce the vibration exposure but maintained the transmissibility ratio from the vehicle to user. It was worth noting that the VDV results were below the lower limit of an eight-hour exposure (0.1 m/s^1.75^). Wheelchair docking systems should aim to secure the wheelchair in a vehicle to minimize high accelerations (shocks), therefore, reducing VDV effects and user’s discomfort for a short time of shock exposure. RMS vibration reduction can be complemented with different wheelchair cushions [26] and EPW suspension [27]; however, further analysis is needed to isolate these factors.

Aim 2 and 3 examined the usability of the QLX prototype compared to participants’ WDS (i.e., 4-TD and EZ-Lock). Participants rated higher usability scores and better driving experience when using the wheelchair with the QLX compared to their own WDS, but the differences were not statistically different except when docking the QLX in the vehicle. Given that most of the participants used 4-TD as their WDS, these are positive findings as participants required minimum training to use the study wheelchair with QLX and independently docked the wheelchair to the vehicle on their own. The wheelchair driving test also aimed to analyze driving limitations with the QLX, which has a low-profile docking system. Studies showed that wheelchair users who use EZ-Locks reported discomfort as the docking pin scratches the floor when passing over a pothole or uneven surface [10], which could damage the wheelchair and injure the user [11]. Wheelchairs that use a 4-TD did not affect their ground clearance; therefore, participants did not have any difficulty passing over uneven surfaces. Likewise, the QLX showed similar performance to the 4-TD docking system, indicating that the QLX had sufficient ground clearance to avoid problems encountered when performing different driving tasks.

Participants reported no significant difference in task load demand between WDS when riding in a vehicle. In general, wheelchair users who use 4-TD require assistance from caregivers, travel companions, or transit drivers to engage the 4-TD for them. Thus, participants report little to no task load demand when using this docking system. On the other hand, participants reported similar low task load demand results when using the QLX by themselves. This finding indicates that using the QLX may be as easy as using a 4-TD system that requires wheelchair users to exert minimal effort.

### 4.1. Limitations

The unfamiliarity with the study wheelchair with QLX may have affected the participants’ perception toward the use of the QLX. Participants reported years of experience using power wheelchairs, yet their driving performance to dock the QLX in the vehicle may be influenced by the drive wheel configuration, speed parameters, and seating configuration. The study wheelchair was configured as similar as possible to the participants’ own wheelchairs to minimize these challenges. Another limitation was the limited variety of WDS evaluated in the study. Although most of the participants were using 4-TD, a small number of them were using EZ-Lock. Therefore, the usability comparison was performed with and without the EZ-Lock. However, it was not included in the vibration study because it offers similar rigidity as the QLX prototype. A third limitation is that responses to comfort and safety questionnaires showed a ceiling effect which may have decreased the ability to find significant differences between docking systems. This could be resolved by recruiting a larger sample size. Qn the other hand, no significant differences in the use of different WDS demonstrate the ease of use to operate the QLX independently. Last, the vehicle driving speed was ≤25 mph due to IRB restrictions and traffic laws in the testing area; therefore, WBV exposure should be further explored at higher speeds and more varied driving activities.

### 4.2. Suggestions for Future Research

Since it was a prototype, due to IRB restrictions and user safety, it was practically impossible to test both usability and WBV simultaneously while riding in a vehicle over real-world surfaces. While the vibration test (Aim 1) and vehicle riding test (Aim 3) used similar driving tasks, it is not possible to provide a conclusive relationship between WBV exposure and the user’s discomfort with WDS while riding a vehicle. We suggest future research to evaluate this relationship with WDS in different real-world environments (e.g., residential, city) to improve WBV exposure the IS02631-1, as acceptable values of vibrations magnitude for comfort vary per application. In addition, although this study tries to control for external factors that may affect the vibration transmissibility ratio by using the same vehicle driven by the same driver, there may be other factors that we were not able to control. Therefore, future research may need to consider the vehicle vibration criteria from the chassis vibration to the wheelchair seat vibration.

## 5. Conclusions

The study performed a systematic evaluation of WBV effects and usability of the QLX prototype, a low-profile automatic-powered WDS. To our knowledge, this is the first study to analyze WBV exposure of wheelchair users using WDS while riding in vehicles. Results showed WDS rigidity to minimize shocks and reduce discomfort. Vibration exposure with the testing WDS was higher than the health-risk zone; however, external factors such as vehicle settings and the selection of extreme road surface conditions likely influenced the exposure. In addition, the QLX prototype received positive ratings from wheelchair users while driving on common wheelchair driving tasks and while riding in a vehicle. Results showed similar comfort, safety, and ease of use to 4-TD, with the main difference of no assistance needed to dock the wheelchair to the vehicle, therefore, enhancing user autonomy Overall, the results suggest the QLX is an easy, quick, safe, and preferred docking system for wheelchair users.

## Supplementary Material

2

## Figures and Tables

**Table 1.** Descriptive of study participants.

#	Gender	Age	Type of Disability	Years of Using Wheelchair	Wheelchair Locking System	Vehicle Riding Test

1	F	35–54	Physical/Mobility	10+	TD	TD + SV
2	F	35–54	Spinal cord injury	10+	EZ	TD + SV
3	F	35–54	Physical/Mobility + Invisible	4–6	TD	TD + SV
4	M	55–64	Physical/Mobility + Hearing	7–10	TD	TD + SV
5	F	35–54	Spinal cord dysfunction	4–6	TD	TD + SV
6	M	35–54	Spinal cord injury	10+	EZ	EZ + PV
7	M	27–34	Spinal cord injury + Spinal cord dysfunction	4–6	TD	TD + SV
8	F	35–54	Physical/Mobility	10+	TD	TD + PV
9	M	35–54	Spinal cord injury Physical/Mobility + Head injury	10+	TD	TD + PV
10	F	65+	+ Vision + Hearing + Psychological	7–10	TD	DS + PV
11	M	27–34	Spinal cord injury	7–10	TD	TD + SV
12	M	35–54	Physical Mobility + Spinal cord injury	10+	TD	TD + SV
13	M	65+	Other	10+	TD	TD + SV
14	M	65+	Physical/Mobility	10+	EZ	DS + PV
15	F	55–64	Physical/Mobility + Spinal cord injury	10+	TD	TD + SV

4-TD = 4-point tiedown, EZ = EZ-Lock, DS = Driving in the driver’s seat, SV = Study vehicle, PV = Personal vehicle.

**Table 2.** Wheelchair driving test results (n = 15).

	Wheelchair with QLX		Participants’ Personal WDS		*p*

	M (SD)	Median (IQR1–3)	M (SD)	Median (IQR1–3)	

sus	80.9 (13.3)^a^	81.3 (70–92.5)	70.7 (25.5)	72.5(50–97.5)	0.12
Driving Experience					
Curb cut	8.5 (2.0)	10 (8–10)	7.3 (2.8)	9 (5–10)	0.31
Grass	8.9 (1.7)	10 (8–10)	8.3 (1.9)	9 (7–10)	0.27
Uneven sidewalk	8.1 (2.5)	9(7–10)	6.9 (2.6)	7 (4–10)	0.19
Potholes	5.3 (3.0)	5(4–7)	4.7 (3.0)	5(1–7)	0.76
Vehicle ingress/egress	8.7(1.1)	9 (8–10)	8.1 (1.9)	9(7–10)	0.27
Docking in vehicle	8.5 (2.1)	9 (8–10)	5.9 (2.8)	5 (5–8)	0.03*
Overall	8.7 (1.8)	9 (8–10)	7.5 (2.0)	7 (5–10)	0.07

*p* < 0.05.

a One participant did not respond.

**Table 3.** Vehicle riding test results (n = 15).

	Wheelchair with QLX		Participants’ Personal WDS		*p*
	M (SD)	Median (IQR1–3)	M (SD)	Median (IQR1–3)	
SUS	86.5 (13.9)	90 (75–100)	58.8 (22.6)	52.5 (42.5–75)	0.01 **
NASA TLX					
Mental demand	17.3 (19.4)	10 (5–20)	22.0 (26.0)	5 (5–35)	0.63
Physical demand	18.7 (20.3)	10 (5–30)	27.7 (26.9)	20 (5–55)	0.78
Temporal demand	11.7 (10.6)	5 (5–15)	24.7 (26.6)	10 (5–55)	0.07
Performance	20.7 (26.4)	10 (5–15)	22.3 (26.7)	15 (5–25)	0.49
Effort	19.0 (17.7)	15 (5–30)	29.7 (30.3)	15 (5–60)	0.11
Frustration	15.7(17.0)	10 (5–15)	25.3 (28.1)	10 (5–45)	0.88
Comfort questionnaire					
Riding	9.3 (0.9)	10 (9–10)	8.1 (2.2)	9 (6–10)	0.02*
Docking in the vehicle	9.1 (0.9)	9 (9–10)	6.9 (3.1)	7 (5–10)	0.03*
Vehicle ingress/egress	9.0 (1.3)	9 (9–10)	8.0 (1.8)	8 (7–10)	0.03*
Overall	9.3 (1.0)	10 (9–10)	8.1 (2.1)	9 (6–10)	0.02*
Security questionnaire					
Accelerating/Starting the vehicle	9.3 (1.0)	10 (9–10)	8.2 (2.5)	9 (8–10)	0.06
Decelerating/Stopping the vehicle	9.3 (0.9)	10 (9–10)	8.2 (2.5)	9(8–10)	0.03*
Turning	9.3 (1.0)	10 (9–10)	7.9 (2.7)	9(7–10)	0.01*
Riding	9.4 (0.9)	10 (9–10)	8.5 (2.1)	10 (8–10)	0.02*
Overall	9.3 (1.1)	10 (9–10)	8.3 (2.3)	10 (7–10)	0.02*

* *p* < 0.05,

** *p* < 0.01.

## Data Availability

The data presented in this study are available on request from the corresponding author. The data are not publicly available due to privacy restrictions.

## Highlights:

### What are the main findings?

- The QLX showed slightly lower whole-body vibration and shocks exposure than the 4-point tiedown system while riding the vehicle.
- The QLX showed better usability, especially when docking in the vehicle, than other wheelchair docking systems.

### What is the implication of the main finding?

- The QLX has sufficient ground clearance to avoid the problems commonly encountered when traversing uneven surfaces.

## References

[1] Disability Characteristics. 2022. Available online: https://data.census.gov/table?q=Disability+characteristics (accessed on 7 October 2022).

[2] Travel Patterns of American Adults with Disabilities. 2018. Available online: https://www.bts.dot.gov/sites/bts.dot.gov/files/docs/explore-topics-and-geography/topics/passenger-travel/222466/travel-patterns-american-adults-disabilities-11-26-19.pdf (accessed on 7 October 2022).

[3] Lee CD; Koontz AM; Cooper R; Sivakanthan S; Chemicoff W; Brunswick A; Deepak N; Kulich HR; Laferrier J; Collins NL; Understanding Travel Considerations and Barriers for People with Disabilities to Using Current Mode of Transportation through Journey Mapping. Transp. Res. Rec. 2022; Submitted. 10.1177/03611981231188730 PMC11271742 39055859

[4] Klinich KD; Manary MA; Orton NR; Boyle KJ; Hu J. A literature review of wheelchair transportation safety relevant to automated vehicles. Int. J. Environ. Res. Public Health 2022,19,1633. 35162657 10.3390/ijerph19031633 PMC8835052

[5] Buning ME; Bertocci G; Schneider LW; Manary M; Karg P; Brown D; Johnson S. RESNA’s position on wheelchairs used as seats in motor vehicles. Assist. Technol. 2012,24,132–141. 22876735 10.1080/10400435.2012.659328

[6] Van Roosmalen L; Orton NR; Schneider L. Safety, usability, and independence for wheelchair-seated drivers and front-row passengers of private vehicles: A qualitative research study. /. Rehabil. Res. Dev. 2013,50,239. 10.1682/jrrd.2011.11.0217 23761005

[7] Perez B; Choi J; Paquet V; Lenker J; Kocher L; Nemade M; Kern C; Steinfeld E. Comparison of wheelchair securement systems designed for use in large accessible transit vehicles (LATVs). Assist. Technol. 2021,33,105–115. 31070523 10.1080/10400435.2019.1604582

[8] Van Roosmalen L; Karg P; Hobson D; Turkovich M; Porach E. User evaluation of three wheelchair securement systems in large accessible transit vehicles. J. Rehabil. Res. Dev. 2011,48,823–838. 21938667 10.1682/jrrd.2010.07.0126

[9] Hryciow Z. The safety of wheelchair occupants in motor vehicles. Arch. Motoryz. 2022,97,5–13.

[10] Philips GR; Clark C; Wallace J; Coopmans C; Pantic Z; Bodine C. User-centred design, evaluation, and refinement of a wireless power wheelchair charging system. Disabil. Rehabil. Assist. Technol. 2022,17,815–827. 32924672 10.1080/17483107.2020.1818135

[11] Hogaboom NS; Worobey LA; Houlihan BV; Heinemann AW; Boninger ML. Wheelchair breakdowns are associated with pain, pressure injuries, rehospitalization, and self-perceived health in full-time wheelchair users with spinal cord injury. Arch. Phys. Med. Rehabil. 2018,99,1949–1956. 29698640 10.1016/j.apmr.2018.04.002

[12] Qatu M. Recent research on vehicle noise and vibration. Int. f. Veh. Noise Vib. 2012,8,289.

[13] Singh GK. Effect of whole-body vibration on vehicle operators: A review. Int. }. Sci. Res. 2014,3,320–323.

[14] Jee S-H; Yi J-C. The Application of the Simulation Techniques to Reduce the Noise and Vibration in Vehicle Development; SAE Technical Paper; SAE: Warrendale, PA, USA, 2000.

[15] ISO 2631-1; Mechanical Vibration and Shock-Evaluation of Human Exposure to Whole-Body Vibration—Part 1: General Requirements. The International Standards Organization (ISO): Geneva, Switzerland, 1997.

[16] Cardinale M; Pope MH. The effects of whole body vibration on humans: Dangerous or advantageous? Acta Physiol. Hung. 2003,90,195–206. 14594190 10.1556/APhysiol.90.2003.3.2

[17] Griffin M. Discomfort from feeling vehicle vibration. Veh. Syst. Dyn. 2007,45,679–698.

[18] Chwalik-Pilszyk G; Ziemianski D; Kozien MS. Experimental investigations on transmission of whole body vibration to the wheelchair user’s body. Open Eng. 2022,12,431–438.

[19] Brooke J. SUS-A quick and dirty usability. Usability Eval. Ind. 1996,189,4r–7.

[20] Lewis JR; Sauro J. The factor structure of the system usability scale. In International Conference on Human Centered Design; Springer: Berlin/Heidelberg, Germany, 2009; pp. 94–103.

[21] Hart SG; Staveland LE. Development of NASA-TLX (Task Load Index): Results of empirical and theoretical research. In Advances in Psychology; Elsevier: Amsterdam, The Netherlands, 1988; Volume 52, pp. 139–183.

[22] Battiste V; Bortolussi M. Transport pilot workload: A comparison of two subjective techniques. In Proceedings of the Human Factors Society Annual Meeting; SAGE Publications Sage CA: Los Angeles, CA, USA, 1988; Volume 32, pp. 150–154.

[23] Rubio S; Diaz E; Martin J; Puente JM. Evaluation of subjective mental workload: A comparison of SWAT, NASA-TLX, and workload profile methods. Appl. Psychol. 2004,53,61–86.

[24] MathWorks. MATLAB R2022b; MathWorks: Natick, MA, USA, 2022.

[25] StataCorp. Stata Statistical Software: Release 16; StataCorp LLC: College Station, TX, USA, 2019.

[26] Garda-Mendez Y; Boninger ML. Dynamic stiffness and transmissibility of commercially available wheelchair cushions using a laboratory test method. J. Rehabil. Res. Dev. 2012,49, 7. 22492334 10.1682/jrrd.2011.02.0023

[27] Candiotti JL; Neti A; Sivakanthan S; Cooper RA. Analysis of Whole-Body Vibration Using Electric Powered Wheelchairs on Surface Transitions. Vibration 2022,5,98–109. 35434527 10.3390/vibration5010006 PMC9009286

