# Supplementary material for: Usability and Vibration Analysis of a Low-Profile Automatic Powered Wheelchair to Motor Vehicle Docking System
Source: Vibration. Author manuscript; Available in PMC 2023 Oct 26. (PMC10601336; doi:10.3390/vibration6010016)
Supplement: 2 [file NIHMS1929414-supplement-2.pdf]

vibration

MDPI

Table S1: Wheelchair Driving Experience Questionnaire

Please rank the following questions on a scale of 0-10 (0-Baad experience, 10-Good experience) by filling in the circle that is most representative of your experience.

1. How was your experience when ascending/descending the curb cut?

Bad experience (0)	1	2	3	4	Neutral experience (5)	6	7	8	9	Good experience (10)
◦	◦	◦	◦	◦	◦	◦	◦	◦	◦	◦

2. How was your experience when driving through grass?

Bad experience (0)	1	2	3	4	Neutral experience (5)	6	7	8	9	Good experience (10)
◦	◦	◦	◦	◦	◦	◦	◦	◦	◦	◦

3. How was your experience when driving over the uneven sidewalk?

Bad experience (0)	1	2	3	4	Neutral experience (5)	6	7	8	9	Good experience (10)
◦	◦	◦	◦	◦	◦	◦	◦	◦	◦	◦

4. How was your experience when driving over potholes?

Bad experience (0)	1	2	3	4	Neutral experience (5)	6	7	8	9	Good experience (10)
◦	◦	◦	◦	◦	◦	◦	◦	◦	◦	◦

5. How was your experience when entering/exiting the vehicle?

Bad experience (0)	1	2	3	4	Neutral experience (5)	6	7	8	9	Good experience (10)
◦	◦	◦	◦	◦	◦	◦	◦	◦	◦	◦

6. How was your experience when docking in the vehicle?

Bad experience (0)	1	2	3	4	Neutral experience (5)	6	7	8	9	Good experience (10)
◦	◦	◦	◦	◦	◦	◦	◦	◦	◦	◦

7. How was your experience overall?

Bad experience (0)	1	2	3	4	Neutral experience (5)	6	7	8	9	Good experience (10)
○	○	○	○	○	○	○	○	○	○	○

Table S2: Comfort Questionnaire

Please rank the following questions on a scale of 0-10 (0-Not at all comfortable, 10-Extremely comfortable) by filling in the circle that is most representative of your experience.

**1. How comfortable did you feel when riding in the vehicle?**										
Not at all comfortable (0)	1	2	3	4	Moderately comfortable (5)	6	7	8	9	Extremely comfortable (10)
○	○	○	○	○	○	○	○	○	○	○
**2. How comfortable did you feel when docking in the vehicle?**										
Not at all comfortable (0)	1	2	3	4	Moderately comfortable (5)	6	7	8	9	Extremely comfortable (10)
○	○	○	○	○	○	○	○	○	○	○
**3. How comfortable did you feel when entering/exiting the vehicle?**										
Not at all comfortable (0)	1	2	3	4	Moderately comfortable (5)	6	7	8	9	Extremely comfortable (10)
○	○	○	○	○	○	○	○	○	○	○
**4. How comfortable did you feel overall?**										
Not at all comfortable (0)	1	2	3	4	Moderately comfortable (5)	6	7	8	9	Extremely comfortable (10)
○	○	○	○	○	○	○	○	○	○	○

Table S3: Security Questionnaire

Please rank the following questions on a scale of 0-10 (0-Not at all secure, 10-Extremely secure) by filling in the circle that is most representative of your experience.

**1. How secure did you feel when the vehicle was accelerating/starting?**										
Not at all secure (0)	1	2	3	4	Moderately secure (5)	6	7	8	9	Extremely secure (10)

○	○	○	○	○	○	○	○	○	○	○
**2. How secure did you feel when the vehicle was decelerating/stopping?**										
Not at all secure (0)	1	2	3	4	Moderately secure (5)	6	7	8	9	Extremely secure (10)
○	○	○	○	○	○	○	○	○	○	○
**3. How secure did you feel when the vehicle was turning?**										
Not at all secure (0)	1	2	3	4	Moderately secure (5)	6	7	8	9	Extremely secure (10)
○	○	○	○	○	○	○	○	○	○	○
**4. How secure did you feel when riding in the vehicle?**										
Not at all secure (0)	1	2	3	4	Moderately secure (5)	6	7	8	9	Extremely secure (10)
○	○	○	○	○	○	○	○	○	○	○
**5. How secure did you feel overall?**										
Not at all secure (0)	1	2	3	4	Moderately secure (5)	6	7	8	9	Extremely secure (10)
○	○	○	○	○	○	○	○	○	○	○

Table S3: Vehicle riding test results, excluding responses of participants who did not use 4-point tiedown. (n=12)

	Wheelchair with QLX		Participants' personal WDS		*p*
	M (SD)	Median (IQR1-3)	M (SD)	Median (IQR1-3)	
SUS	78.1 (12.2)	77.5 (68.8-87.5)	68.1 (25.7)	70 (47.5-93.75)	0.24
NASA TLX					
Mental demand	15.0 (18.1)	7.5 (5-12.5)	20.0 (22.4)	7.5 (5-32.5)	0.63
Physical demand	13.8 (11.3)	7.5 (5-27.5)	22.1 (20.4)	15 (5-32.5)	0.06
Temporal demand	12.1 (11.4)	7.5 (5-12.5)	25.4 (27.3)	12.5 (5-47.5)	0.13
Performance	16.7 (20.3)	10 (5-15)	22.0 (22.1)	15 (5-20)	0.19
Effort	16.7 (13.5)	12.5 (5-25)	29.2 (27.9)	15 (5-57.5)	0.05*
Frustration	12.9 (8.4)	10 (7.5-15)	22.1 (26.1)	10 (5-30)	0.94
Comfort questionnaire					
Riding	9.3 (1.0)	9.5 (9-10)	8.1 (2.3)	9 (6-10)	0.06

Docking in the vehicle	9.1 (1.0)	9 (8.5-10)	6.7 (3.1)	7 (5-10)	0.03*
Vehicle Ingress/Egress	8.9 (1.4)	8.5 (8-9)	8.1 (1.7)	8.5 (7-9.5)	0.10
Overall	9.2 (1.0)	9.5 (8.5-10)	8.1 (2.2)	8.5 (6.5-10)	0.06
Security questionnaire					
Accelerating/Starting the vehicle	9.2 (1.0)	9.5 (8.5-10)	7.8 (2.7)	9 (6.5-10)	0.06
Decelerating/Stopping the vehicle	9.3 (1.0)	9.5 (9-10)	7.9 (2.7)	9 (6.5-10)	0.06
Turning	9.3 (1.1)	10 (8.5-10)	7.5 (2.9)	8.5 (5.5-10)	0.01**
Riding	9.3 (1.0)	10 (9-10)	8.3 (2.3)	9 (7-10)	0.03*
Overall	9.3 (1.2)	10 (9-10)	8.1 (2.5)	9 (6.5-10)	0.03*

*$p<.05$, **$p<.01$
